# Early Risk Detection of Burnout: Development of the Burnout Prevention Questionnaire for Coaches

**DOI:** 10.3389/fpsyg.2019.00714

**Published:** 2019-04-05

**Authors:** Paul Schaffran, Jens Kleinert, Sebastian Altfeld, Christian Zepp, Konrad Wolfgang Kallus, Michael Kellmann

**Affiliations:** ^1^Unit of Sport Psychology, Faculty of Sport Science, Ruhr University Bochum, Bochum, Germany; ^2^Institute of Psychology, German Sport University Cologne, Cologne, Germany; ^3^German Research Centre for Elite Sport Cologne, German Sport University Cologne, Cologne, Germany; ^4^Institute of Psychology, University of Graz, Graz, Austria; ^5^School of Human Movement and Nutrition Sciences, University of Queensland, Brisbane, QLD, Australia

**Keywords:** emotional exhaustion, coaching, stress, recovery, motivation

## Abstract

**Introduction:** Previous research has shown that burnout develops as the result of a continuous imbalance between chronic stress and appropriate coping resources. Hence, the essential factors to measure burnout encompassed the factors stress and recovery within our studies. However, the Burnout Prevention Questionnaire for Coaches (BPQ-C) does not represent a new questionnaire from scratch, but rather a re-evaluated, condensed, and subsequently combined instrument with scales derived from validated psychometric instruments.

**Methods:** The objective of study 1 (*N* = 233) was to create and evaluate the psychometric structure of the BPQ-C. The aim of study 2 (*N* = 473) consisted in the validation of the BPQ-C via a Confirmatory Factor Analysis.

**Results:** The Exploratory Factor Analysis resulted in a model with three dimensions (Pre-Burnout, Resources, and Burnout). Via the subsequent Confirmatory Factor Analysis, the model could be confirmed with good fit indices (χ2 = 96.898, *df* = 19, *p* < 0.001, CFI = 0.973, SRMR = 0.044, RMSEA = 0.093, LO90 = 0.075, HI90 = 0.112).

**Conclusion:** The BPQ-C includes a number of previously established risk and protective factors within a single psychometric instrument. The systematic application of the BPQ-C can help to detect critical conditions at an early stage in order to derive individualized and beneficial interventions for the respective coaches.

## Introduction

In the context of a growing sport-scientific interest in burnout in sports, the coaches' perspective is seldom represented. Furthermore, the majority of the published studies about burnout discuss the athletes' perspectives (McKay et al., 2008; Lonsdale et al., 2009). However, compared to athletes, coaches have to deal with a multifarious range of responsibilities (Fletcher and Scott, 2010). Apart from teaching technical and tactical skills, they also support the personal and social development of their athletes. In addition, coaches have to cope with several adverse circumstances, such as inconvenient working hours (e.g., morning, night and/or weekend training), a high workload, traveling, temporary contracts, job insecurity, role conflicts, and media pressure (Dixon and Bruening, 2005; Olusoga et al., 2010; Lundkvist et al., 2012). Especially the working environment of high-performance coaches can be characterized as complex, dynamic, and turbulent due to its unpredictability, variability, and competitiveness (Rynne et al., 2006; Fletcher and Scott, 2010). Being affected by this potpourri of straining internal and external stressors may ultimately lead to a chronic stress reaction.

Following Lazarus's (1999) transactional model of stress and coping, several authors assume that burnout can occur as one negative outcome of prolonged stress associated with the interaction of personal and environmental factors (Smith, 1986; Kelley and Baghurst, 2009), which is resulting in a continuous imbalance between chronic stress and appropriate coping resources (Kelley and Gill, 1993; Smith, 2007). In this context, Kellmann (2002) has introduced the ‘scissor model’ of the interrelation between stress states and recovery demands. The theoretical framework states that with increasing stress, increasing recovery is necessary. According to the model, coaches (as well as athletes) can compensate short-term stress without additional recovery interventions. However, continuous inadequate or lacking recovery activities before or after stressful situations can lead to deficits in coping with emotional, physical, and psychological reactions (Kallus and Kellmann, 2016). According to these theoretical deliberations, Kallus (2016b) suggests that burnout can be defined as a person's state of maximum need of recovery. The correlation of stress, recovery, and burnout could be proved in quantitative as well as qualitative studies with coaches. Regression analyses by Altfeld and Kellmann (2015), e.g., showed that recovery and stress revealed the only significant effects on the dependent variable *emotional exhaustion* in a model with the additional independent variables age, working hours, financial security, feeling to meaningfulness, feeling of success, and sense of well-being. A longitudinal study conducted by Bentzen et al. (2017) included coaches with low and high burnout values, who were evaluated throughout a whole season, as participants. This quantitative-qualitative mixed-method approach showed that differences in the burnout degree could be explained by looking at the motivational profile, work-home interference, and the ability to meet recovery demands.

However, a universal definition of burnout does not exist up to the present (Schaffran et al., 2016). Sport-related research (especially in coaches) has primarily adapted the widely accepted concept proposed by (Maslach et al., 1996). Following this approach, burnout is characterized by three symptoms: emotional exhaustion, cynism (depersonalization), and reduced sense of personal accomplishment (Maslach et al., 1996). Emotional exhaustion describes the key symptom of burnout and delineates the most obvious manifestation of this complex syndrome (Maslach et al., 2001). Moreover, it represents the most widely reported and most thoroughly analyzed aspect of burnout (Schaffran et al., 2016). The development of burnout portrays a process in time that may last several months or even years (Leiter and Maslach, 1988; Lee and Ashforth, 1996; Taris et al., 2005). Maslach and Leiter (2008) as well as the meta-analysis about the correlation of the three burnout symptoms as described by Lee and Ashforth (1996) show that emotional exhaustion manifests first in the development of burnout. Depersonalization and a reduced sense of personal accomplishment emerge from this symptom.

The assessment of burnout is usually realized using self-report questionnaires (Altfeld and Kellmann, 2013). In this context, the *Maslach Burnout Inventory* (Maslach et al., 1996) exemplifies the most frequently implemented questionnaire. The MBI includes 22 items divided into the three scales emotional exhaustion, depersonalization, and personal accomplishment. Apart from the MBI as a general burnout measuring tool, Harris and Ostrow (2008) have created the *Coach Burnout Questionnaire* (CBQ) to assess burnout as a coach-specific instrument, which also focuses on the three central dimensions of burnout proposed by (Maslach et al., 1996). According to the MBI and the CBQ, high values in emotional exhaustion and depersonalization and low values in personal accomplishment indicate a high risk of burnout (Maslach et al., 1996; Harris and Ostrow, 2008). However, Burisch (2013) indicates that these questionnaires may not be applicable in field conditions as they do not offer guidance and recommendations for practical contexts. He criticizes that a coach suffering from burnout is forced into a long break, which potentially includes therapeutic treatment as well. Primary burnout prevention cannot be realized using the MBI and CBQ due to the lengthy evaluation procedure (Burisch, 2013). Regardless of the fact that during the last decades an increased number of international studies had focused on correlates of burnout, sensitive and valid measurements for the identification of risk factors of burnout in coaches are still missing (Fletcher and Scott, 2010).

At least three perspectives for the development of burnout emerge from existing concepts. Self-determination theory (SDT; Ryan and Deci, 2002) is an approach to human motivation and personality. According to the SDT, the satisfaction of basic psychological needs (autonomy, competence, relatedness) is associated with a positive development of the individual and with fulfilling and meaningful behavior. This leads to an increased mental well-being and serves as a prevention against burnout (Reinboth and Duda, 2006). A multidimensional understanding of motivation is the basis for the SDT and can be categorized along a continuum of self-determination. Amotivation (i.e., behavior without understanding sense and meaning of it) marks one end of the continuum. Extrinsic motivation, which can be divided into four different types of behavior regulation can differ in their degree of autonomy. This includes two types of controlled and little autonomous behavior: external (e.g., motivated by fear) and introjected (e.g., standard/regulation that is bound to standards) regulation (Ryan and Deci, 2002). The remaining two types rather describe autonomous and strongly internally regulated behavior: identified (i.e., bound to reason) and integrated (i.e., bound to identity) regulation. The other end of the continuum shows an intrinsic motivation that refers to interesting and enjoyable types of behavior that are engaged in freely and out of choice. Following this model, the development of burnout is all the more probable, the less internalized or intrinsic the reasons and motives for coaches activities are (McLean and Mallett, 2012).

A second approach focuses on different areas of work life, which imply a broad range of psycho-bio-social mechanisms on the development of burnout (Leiter and Maslach, 2004). According to this approach, the following six areas of job-person mismatch are the critical factors for a development of burnout: *workload, control, reward, community, fairness*, and *values*. Leiter and Maslach (2004) postulate that the development of burnout correlates directly with an increasing *workload*. When the workload of the job exceeds the individual resources, emotional exhaustion is a common result. *Control* encompasses the (perceived) ability of a person to influence work-related decisions and accordingly have a personal autonomy (Brom et al., 2015). Hence, *control* works protectively against the development of burnout. The *reward* area of work life considers the extent to which the rewards (monetary, social, intrinsically) agree with the expectations of a person. A smaller agreement means a lower identification with the objectives of the organization, which increases the risk of developing burnout (Leiter and Maslach, 2004). *Community* comprises social interactions at work. Support from the family, friends, or coaching colleagues can function as a protection against burnout (Hendrix et al., 2000). However, conflicts in these contexts (private or work-related) can have a negative influence on burnout as well (Mazerolle et al., 2008). *Fairness* describes the extent to which a person perceives decisions at work as fair and feels treated with respect. Mutual respect again is a pivotal element in order to receive a sense of community (Leiter and Maslach, 2004). The area of *values* is one of the main aspects of a coach's relation to his work. It comprises the ideals and motivations which initially made the job attractive to the coach and can be described as the motivating connection between a coach and his club, which is more than just an exchange of time for money. This approach does not focus on the consideration of burnout as a person-related problem. Instead, the coincidence between traits of a job/work and the person serves as a precondition for the development of burnout.

The third approach combines chronic stress states with underrecovery (Kellmann, 2002). In this context, burnout is understood as a person's maximum need for recovery (Kallus, 2016b). Therefore, burnout emerges when recovery means cannot compensate the stress over a longer period of time.

As the development of burnout seems to be multifaceted, a screening instrument for burnout prevention should incorporate the different possible psychological “pathways” of burnout development and maybe try to weigh them. Emotional exhaustion would be a good candidate for validation as it is agreed to be the first burnout symptom to appear (Lee and Ashforth, 1996). Burnout as a clinical syndrome is closely associated with depressive mood. However, the distinction between burnout and (reactive) depression is still an unresolved issue in the clinical treatment of burnout (Ahola et al., 2005). Nonetheless, mood states are closely interlinked to burnout and constitute a basic element of burnout symptoms. Thus, changes in psychological well-being are a sensitive facet in the development of burnout.

The aim of this research hence consisted in the synthesis of a questionnaire to measure risk factors of burnout in coaches. In detail, the aim of our studies was to incorporate the different approaches into a new, economic questionnaire. We did not develop a new instrument from scratch, as the concepts of stress, recovery, areas of worklife, and motivation can be assessed via validated and reliable assessment tools. In effect, we re-evaluated, condensed, and subsequently combined scales of validated psychometric instruments into the Burnout Prevention Questionnaire for Coaches (BPQ-C): a new tool for the early detection of risk factors to prevent the development of burnout in coaches.

## Study 1

The objective of study 1 was to create and evaluate the psychometric structure of the BPQ-C in two steps. First, regression analyses with all scales of the selected questionnaires were conducted to determine which scales contributed most to the central aspects of stress, recovery, motivational imbalance, and work-related risk factors for burnout. This step aimed at the identification of relevant scales with regard to risk factors for burnout in coaches. In a second step, all extracted scales were checked for a possible overlap with an Exploratory Factor Analysis to obtain a factor structure for the BPQ-C.

### Method

#### Sample

A total of 233 German coaches (64 females) with a mean age of 37.32 years (*SD* = 13.58 years) participated in this study. The numerical superiority of male coaches within this study corresponds to the distribution of female and male coaches in Germany (Bahlke et al., 2003; Altfeld and Kellmann, 2015). Full-time (*n* = 54), part-time (*n* = 76), and voluntary coaches (*n* = 103) were represented, with a relatively even distribution of coaches in team (*n* = 129) and individual sports (*n* = 104). Active support or coaching of athletes in the indicated sport during the time of assessment served as a selection criterion for the participation in the study. Table 1 provides a detailed overview of the demographic characteristics.

**Table 1:** Demographic, job characteristics, and contract characteristics of both studies.

**Characteristics**	**Study 1**	**Study 2**
*N*	233	473
Age [M(SD)]	37.32 (13.58)	44.50 (11.30)
Gender (♂, ♀)	73%, 27%	81%, 19%
**TYPE OF SPORT**		
Team	55%	39%
Individual	45%	61%
**SPORT LEVEL**		
International	12%	22%
Highest national	22%	24%
Second highest national	11%	15%
Third highest national	16%	13%
Others	39%	26%
**FORM OF EMPLOYMENT**		
Full-time coaches	23%	54%
Half-time coaches	33%	24%
Voluntary coaches	44%	22%
**COACHING LICENSE/DEGREE**		
Highest national	8%	16%
Second highest national	15%	46%
Third highest national	30%	23%
Fourth highest national	41%	10%
No license	6%	5%
**EXPERIENCE AS A COACH**		
< 1 year	2%	2%
1–2 years	8%	4%
3–5 years	19%	10%
6–10 years	25%	20%
11–15 years	14%	21%
16–25 years	19%	21%
> 25 years	13%	22%

### Measures

The *Recovery-Stress Questionnaire* (RESTQ-Basic; Kallus, 2016a) systematically assesses the recovery-stress state of a person. It can be used within a timeframe of the past seven days and nights (Jimenez et al., 2016). The recovery-stress state expresses to which extent the person is physically and/or mentally stressed, and whether the person is using individual recovery strategies (Kallus, 2016a). The RESTQ-Basic consists of 48 items which are equally divided into 12 scales (four items per scale). Individuals rate the items retrospectively on a seven-point frequency scale ranging from *never* (0) to *always* (6). The RESTQ-Basic has been applied in various settings with German populations (Heidari et al., 2016; Wagner et al., 2017) and has displayed predominantly very good to satisfactory reliabilities (Kallus, 2016a).

In addition to the RESTQ-Basic, the *Recovery-Stress Questionnaire for Coaches* (RESTQ-Coach; Kellmann et al., 2016b) and the *Recovery-Stress Questionnaire for Work* (RESTQ-Work; Jimenez et al., 2016) include coach-specific and work-specific items to allow a recovery-stress profile for coaches or in and for general work settings, respectively. The RESTQ-Coach contains 76 items (28 additional items to the RESTQ-Basic) divided into seven scales, while the RESTQ-Work consists of 93 items (55 additional items) divided into 14 scales. Good reliability (α > 0.70) for all scales is given in both questionnaires (Jimenez et al., 2016; Kellmann et al., 2016b). The RESTQ-Coach has already been used in various studies with German coaches (Altfeld and Kellmann, 2014a,b, 2015; Altfeld et al., 2015). The RESTQ-Work, on the other hand, has been used in bilingual studies regarding the work context (Jimenez and Dunkl, 2017; Wagner et al., 2017), but it has not been applied on German coaches.

The *Areas of Worklife Survey* (AWS; Leiter and Maslach, 1999) assesses multiple job stressors that contribute to the development of burnout. The AWS contains 29 items which are further divided into six scales: workload (6 items), control (3 items), reward (4 items), community (5 items), fairness (6 items), and values (5 items). Items are rated on a five-point frequency scale ranging from *strongly disagree* (1) to *strongly agree* (5). Leiter and Maslach (2004) presented good reliabilities for all scales of the AWS. With regard to German coaches, the AWS has not been utilized yet, but Brom et al. (2015) have validated it for German-speaking populations.

The *Coach Motivation Questionnaire* (CMQ; McLean et al., 2012) measures the six different stages of motivation in coaches. The CMQ consists of 22 items divided into six scales: amotivation (4 items), external regulation (4 items), introjected regulation (4 items), identified regulation (3 items), integrated regulation (3 items), and intrinsic motivation (4 items). Coaches rate the items on a seven-point Likert scale ranging from *not true at all* (1) to *very true* (7). The CMQ has been validated for German coaches by Zepp et al. (2016) and shows good reliabilities for all scales of the CMQ.

The *World Health Organization Well-being Index* (WHO-5; Bech, 2004) is a screening instrument which captures psychological well-being. The WHO-5 consists of five items which are answered on a six-point frequency scale from *at no time* (0) to *all of the time* (5). The five items are added up into a sum score, which mirrors the mental well-being on a scale ranging from 0 to 25. The WHO-5 has been used in German studies with athletes (Ohlert and Ott, 2017), but no German coaches have been subject to any studies yet.

Based on the German *Maslach Burnout Inventory version for Teachers* (Büssing and Perrar, 1992), Altfeld and Kellmann (2014b) modified the *Maslach Burnout Inventory for Coaches* (MBI-C). The MBI-C consists of 22 items divided into three scales: *Emotional Exhaustion* (9 items), *Depersonalization* (5 items), and *Personal Accomplishment* (8 items). Coaches are instructed to answer on a seven-point frequency scale ranging from *never* (0) to *every day* (6).

To create a standardized item structure as basis for further analyses, the response formats of the AWS and CMQ were adapted to the frequency-based item format of the RESTQ, resulting in seven-point frequency scales [*never* (0) to *always* (6)] for these three questionnaires. However, reliabilities were rechecked after this change in item format.

#### Procedure

The study was approved by the university ethic committee of the home university and was conducted according to the Declaration of Helsinki. Informed consent was given by clicking a button on the first page of the online survey. Information about the study was distributed to the coaches with the support of their respective national sport federation. Sport federations were requested to only contact coaches with the third highest national level of coaching license or lower to save the top coaches for the second study. In total, twenty national and regional sport federations were contacted.

### Data Analysis

For the purpose of weighing the different predictors of burnout multiple linear regression analyses were conducted, using the scale *Emotional Exhaustion* (MBI-Coach), and the sum score of the WHO-5 as dependent variables. *Emotional Exhaustion* as a dependent variable mirrors the core symptom of burnout which initiates the development of depersonalization and reduced personal accomplishment (Lee and Ashforth, 1996). The scales *Emotional Exhaustion* and *Personal Accomplishment* of the RESTQ-Coach as well as the scale *Loss of Meaning* of the RESTQ-Work were not considered for the regression models. These scales were adapted from the Maslach Burnout Inventory and depict symptom-like constructs which are inappropriate as predictors. All other scales of the applied questionnaires were set as independent variables, respectively.

As a next step, an Exploratory Factor Analysis (Principal Axis Factoring with Oblimin Rotation) was performed with the remaining scales to obtain a factor structure. In contrast to Principal Component Analysis (as the most widely used method), the aim of Principal Axis Factoring is not only the reduction of data, but also to reveal any latent variables that cause the manifest variables to covary (Costello and Osborne, 2005). Oblimin Rotation, as an oblique rotation method, has been used due to the fact that from theory the factors are not uncorrelated. In oblique rotation methods, the Oblimin Rotation is the most common choice (Costello and Osborne, 2005). Moreover, Fabrigar et al. (1999) postulate that there is no widely preferred method of oblique rotation as all tend to produce similar results. Parallel Analysis, suggested by Horn (1965) as a method to determine the number of factors, was employed to determine the factor structure. In Parallel Analysis, eigenvalues of the determined factors in randomly simulated data sets are compared to those of the factors in the actual data set. In this process, the focus lies on how many of the factors obtained from the actual data have an Eigenvalue greater than that of the simulative data and accordingly the number of factors is decided. The number of factors at the point where the Eigenvalue in the simulative data is greater than that of the actual data is considered to be significant (Çokluk and Koçak, 2016). All analyses were performed with the Statistical Package for Social Survey (SPSS) version 25.0.

## Results and Discussion

Results of the stepwise multiple linear regression analysis for *Emotional Exhaustion* indicate that 57% of the variance in *Emotional Exhaustion* is explained by *General Stress, Social Stress, Amotivation*, and *Fatigue* [*F*_(4, 228)_ = 79.141, *p* < 0.001] (Table 2). For the WHO-5, 73% of the variance is explained by *Being in Shape, General Well-being, Sleep Quality, Disturbed Breaks*, and *Undisturbed Leisure Time* [*F*_(5, 227)_ = 123.365, *p* < 0.001] (Table 3). In total, multiple linear regression analyses revealed that 9 out of 48 scales (12 scales of the RESTQ-Basic, 7 of the RESTQ-Coach, 14 of the RESTQ-Work, 3 of the CBANS, 6 of the CMQ, and 6 of the AWS) explain a significant amount of the variance in *Emotional Exhaustion* and Well-being (WHO-5). Consequently, the following 9 scales remain as a result of the questionnaire development: *General Stress, Social Stress, Fatigue, General Well-being, Sleep Quality* (RESTQ-Basic), *Disturbed Breaks, Being in Shape* (RESTQ-Coach), *Undisturbed Leisure Time* (RESTQ-Work), and *Amotivation* (CMQ).

**Table 2:** Results from stepwise multiple regression of all scales of the RESTQ-Basic, RESTQ-Coach, RESTQ-Work, AWS, and CMQ on *Emotional Exhaustion* of the MBI-C.

**Steps**	**Measurement**	**Unstandardized coefficient**		**Standardized coefficient**	***p***	***F***	***Adjusted R^**2**^***	***ΔR^**2**^***
		**B**	**SE**	**β**				
1						151.134^***^	0.393	
	(Intercept)	5.635	0.661		0.000			
	General Stress	4.051	0.330	0.629	0.000			
2						109.828^***^	0.484	0.091
	(Intercept)	3.019	0.732		0.000			
	General Stress	2.806	0.360	0.436	0.000			
	Amotivation	1.852	0.286	0.361	0.000			
3						100.008^***^	0.561	0.077
	(Intercept)	0.331	0.793		0.677			
	General Stress	1.406	0.396	0.218	0.000			
	Amotivation	1.837	0.264	0.358	0.000			
	Fatigue	2.254	0.349	0.356	0.000			
4						79.141^***^	0.574	0.013
	(Intercept)	−0.655	0.858		0.446			
	General Stress	0.757	0.455	0.118	0.047			
	Amotivation	1.774	0.261	0.346	0.000			
	Fatigue	2.110	0.348	0.333	0.000			
	Social Stress	1.178	0.424	0.171	0.006			

N = 233; RESTQ, Recovery-Stress Questionnaire; AWS, Areas of Worklife Survey; CMQ, Coach Motivation Questionnaire; B, unstandardized beta; SE, standard error; β, standardized beta; p, significance level; F, F statistics; Adjusted R^2^, adjusted variance; ΔR^2^, change in variance;

*** *p < 0.001*.

**Table 3:** Results from stepwise multiple regression of all scales of the RESTQ-Basic, RESTQ-Coach, RESTQ-Work, AWS, and CMQ on the World Health Organization Wellbeing Index.

**Steps**	**Measurement**	**Unstandardized coefficient**		**Standardized coefficient**	***p***	***F***	***Adjusted R^**2**^***	***ΔR^**2**^***
		**B**	**SE**	**β**				
1						412.547^***^	0.639	
	(Intercept)	4.072	0.523		0.000			
	Being in Shape	3.359	0.165	0.801	0.000			
2						261.615^***^	0.692	0.052
	(Intercept)	1.576	0.623		0.012			
	Being in Shape	2.374	0.218	0.566	0.000			
	General Well-being	1.561	0.246	0.330	0.000			
3						190.498^***^	0.710	0.010
	(Intercept)	0.879	0.630		0.164			
	Being in Shape	2.037	0.228	0.485	0.000			
	General Well-being	1.230	0.253	0.260	0.000			
	Sleep Quality	0.776	0.198	0.197	0.000			
4						150.259^***^	0.720	0.010
	(Intercept)	0.542	0.629		0.390			
	Being in Shape	1.861	0.231	0.444	0.000			
	General Well-being	1.246	0.248	0.263	0.000			
	Sleep Quality	0.627	0.200	0.159	0.002			
	Undisturbed Leisure Time	0.423	0.140	0.126	0.003			
5						123.365^***^	0.725	0.005
	(Intercept)	−1.515	1.106		0.172			
	Being in Shape	1.938	0.232	0.462	0.000			
	General Well-being	1.225	0.246	0.259	0.000			
	Sleep Quality	0.746	0.205	0.189	0.000			
	Undisturbed Leisure Time	0.601	0.159	0.179	0.000			
	Disturbed Breaks	0.466	0.207	0.114	0.025			

N = 233; RESTQ, Recovery-Stress Questionnaire; AWS, Areas of Worklife Survey; CMQ, Coach Motivation Questionnaire; B, unstandardised beta; SE, standard error; β, standardised beta; p, significance level; F, F statistics; Adjusted R^2^, adjusted variance; ΔR^2^, change in variance;

*** *p < 0.001*.

The results of the performed Parallel Analysis suggest only one global factor from these nine scales, as the first factor is the only one that shows a greater Eigenvalue for the actual data compared to the simulative data (Table 4). The Eigenvalue of the simulative data surpasses that of the actual data in the second factor already. Hence, the Parallel Analysis points toward a subordination of the recovery scales (*General Well-being, Sleep Quality, Being in Shape, Undisturbed Leisure Time*) and stress scales (*General Stress, Social Stress, Fatigue, Disturbed Breaks*) from the different versions of the RESTQ within this one factor.

**Table 4:** Parallel Analysis for the sample of study 1.

**Factor**	**Eigenvalues of the actual data**	**Eigenvalues of the simulative data**
1	5.26	1.41
2	0.92	1.27
3	0.78	1.17
4	0.57	1.10
5	0.41	1.03
6	0.34	0.97
7	0.29	0.92
8	0.23	0.85
9	0.20	0.79

*N = 233 in both the actual and simulative data*.

There are various authors, however, that see recovery and stress as two individual constructs (Kenttä and Hassmén, 1998; Kellmann, 2010). Due to this, factor analyses with more than one factor will be suggested as possible solutions. In the solution with two factors (Table 5), there are stress and recovery scales within the same factor (factor 2). Further it shows that the scales *Sleep Quality* and *Social Stress* have strong cross loadings and can therefore not clearly be matched with one of the two factors.

**Table 5:** Exploratory factor analysis for a two factor solution for the sample of study 1.

	**Factor 1**	**Factor 2**
Disturbed Breaks	**0.83**	−0.02
Undisturbed Leisure Time	−**0.80**	0.03
Fatigue	**0.71**	0.12
Social Stress	**0.38**	0.35
General Well-being	0.18	**−0.85**
Being in Shape	−0.23	**−0.61**
General Stress	0.31	**0.60**
Amotivation	0.05	**0.54**
Sleep Quality	0.38	**−0.46**

*N = 233. Oblimin rotation was applied. Main factor loadings are printed in bold type*.

Comparable to the two factor solution, even in the three factor solution, high side-loads can be identified for the scales *Sleep Quality, General Well-being*, and *Being in Shape* (Table 6). However, in contrast to the two factor solution, the analyses show that these three recovery scales have been isolated within one factor (Table 6). Only *Undisturbed Leisure Time* could also be assigned to the recovery scales, but as an inverted scale it has a close proximity to *Disturbed Breaks*. A final decision on the factor structure of the BPQ-C should be realized within the Confirmatory Factor Analyses in Study 2.

**Table 6:** Exploratory Factor Analysis for a three factor solution for the sample of study 1.

	**Factor 1**	**Factor 2**	**Factor 3**
Disturbed Breaks	**0.87**	0.03	−0.04
Fatigue	**0.74**	−0.05	0.07
Undisturbed Leisure Time	**−0.72**	−0.12	0.14
Sleep Quality	−0.37	**0.56**	−0.03
General Well-being	−0.02	**0.64**	−0.40
Being in Shape	−0.45	**0.54**	−0.04
Social Stress	0.08	0.17	**0.84**
General Stress	0.17	−0.14	**0.70**
Amotivation	−0.02	−0.19	**0.50**

*N = 233. Oblimin rotation was applied. Main factor loadings are printed in bold type*.

## Study 2

The aim of study 2 was the validation of the BPQ-C. This was done by examining the exploratory structure of the instrument with an independent sample of sport coaches. For this purpose, a Confirmatory Factor Analysis (CFA) was performed and the construct validity as well as the criterion validity were verified by means of intercorrelations and content-related questionnaires, respectively.

### Method

#### Sample

The sample of study 2 consisted of 473 German coaches (90 females) with a mean age of 44.50 years (*SD* = 11.30 years). More than 50 percent of the subjects work as full-time coaches (*n* = 255) and both team (*n* = 184) and individual (*n* = 289) sports were represented. Compared to study 1, coaches in the current sample displayed higher performance levels and possessed coaching licenses with a higher level than those surveyed in study 1 (Table 1).

#### Measures

##### Risk factors for burnout in coaches

The BPQ-C consists of 36 items divided into 9 scales (8 scales with 4 items, the scale *Amotivation* with 3 items, 1 warm-up item). The scale *Undisturbed Leisure Time* has been inverted into *Disturbed Leisure Time*. Coaches rate the items retrospectively on a seven-point frequency scale ranging from *never* (0) to *always* (6).

##### Burnout

Based on the German Maslach Burnout Inventory version for teachers (Büssing and Perrar, 1992), Altfeld and Kellmann (2014b) modified the MBI for coaches (MBI-C). The MBI-C consists of 22 items divided into three scales: *Emotional Exhaustion* (9 items), *Depersonalization* (5 items), and *Personal Accomplishment* (8 items). Coaches are instructed to answer on a seven-point frequency scale ranging from *never* (0) to *every day* (6).

#### Procedure

In line with study 1, the 20 regional and national sport federations were contacted. In contrast to study 1, sport federations were requested to contact coaches with the second highest national level of coaching license or higher. As it was already the case in study 1, this study was approved by the university ethic committee of the home university, conducted according to the Declaration of Helsinki, and informed consent of the coaches was given by clicking a button on the first page of the online survey.

#### Data Analysis

CFAs were performed to confirm the structure of the BPQ-C. In order to do so, all three models of the Exploratory Factor Analysis from study 1 were checked. To evaluate the model fit, the following fit-indices were calculated: χ^2^ and its *p*-value, *comparative fit index* (CFI), *standardized root mean residual* (SRMR), *root mean square error of approximation* (RMSEA)*, lower limit of the 90%-confidence interval* (LO90) und *upper limit of the 90%-confidence interval* (HI90). In the selection of the fit indices used, the present study followed the recommendations of Beauducel and Wittmann (2005), who refer to the consideration of two criteria. On the one hand, fit indices should be utilized that have been used regularly in psychological research to ensure the comparability of the values with other studies as well as the traceability of the results. On the other hand, Tanaka (1993) postulated that the fit indices should cover the widest possible range of different dimensions (absolute vs. incremental, simplicity vs. complexity, and population vs. sample based). Absolute fit indices evaluate how well an a priori model reproduces the sample data. Incremental fit indices evaluate model fit by comparing a target model with a more restricted, nested baseline model. The SRMR and the RMSEA were selected to represent the absolute fit indices, whereas the CFI were selected to represent the incremental fit indices. Another important aspect of fit indices is whether they adjust for model complexity (the number of free parameters of a model). Indices adjusting for complexity favor the more simple models (Tanaka, 1993). Indices favoring simple models selected for this study was the RMSEA. A third characteristic that was represented in the selected fit indices was whether they are population-based or not. The benefit of population-based fit indices is that they are relatively independent from sampling error (Schermelleh-Engel et al., 2003). The population-based fit indices selected in this study were the RMSEA and the CFI. The fit indices were assessed on the basis of the threshold values of Beauducel and Wittmann (2005; CFI > 0.90, SRMR < 0.10) as well as Hu and Bentler (1999; RMSEA < 0.08). CFAs were performed, using SPSS AMOS, version 25.0.

Intercorrelations of scales as well as the stability of intercorrelations across the two samples have been administered to support construct validity. Furthermore, criterion validity has been estimated by looking at the correlation of the BPQ-C with the MBI-C, as the MBI-C is measuring a content-related construct (burnout).

## Results and Discussion

Initially, the results of the CFA revealed an unsatisfactory fit to all of the three models (Table 7). However, the best fit was found for the three factor solution and therefore, further modifications were made on the basis of the three factor model.

**Table 7:** Confirmatory Factor Analyses for all models for the sample of study 2.

**Model**	***X^**2**^***	***df***	***p***	**CFI**	**SRMR**	**RMSEA**	**LO90**	**HI90**
				**≥ 0.9**	**≤ 0.1**	**≤ 0.08**		
One factor model	667.50	27	0.000	0.772	0.090	0.226	0.211	0.241
Two factor model	418.57	26	0.000	0.862	0.067	0.179	0.164	0.194
Three factor model	241.43	24	0.000	0.924	0.052	0.139	0.123	0.155

*N = 473. CFI, Comparative Fit Index; SRMR, Standardized Root Mean Residual; RMSEA, Root Mean Error of Approximation; LO90, lower limit of the 90%-confidence interval; HI90, upper limit of the 90%-confidence interval*.

**Figure 1:**
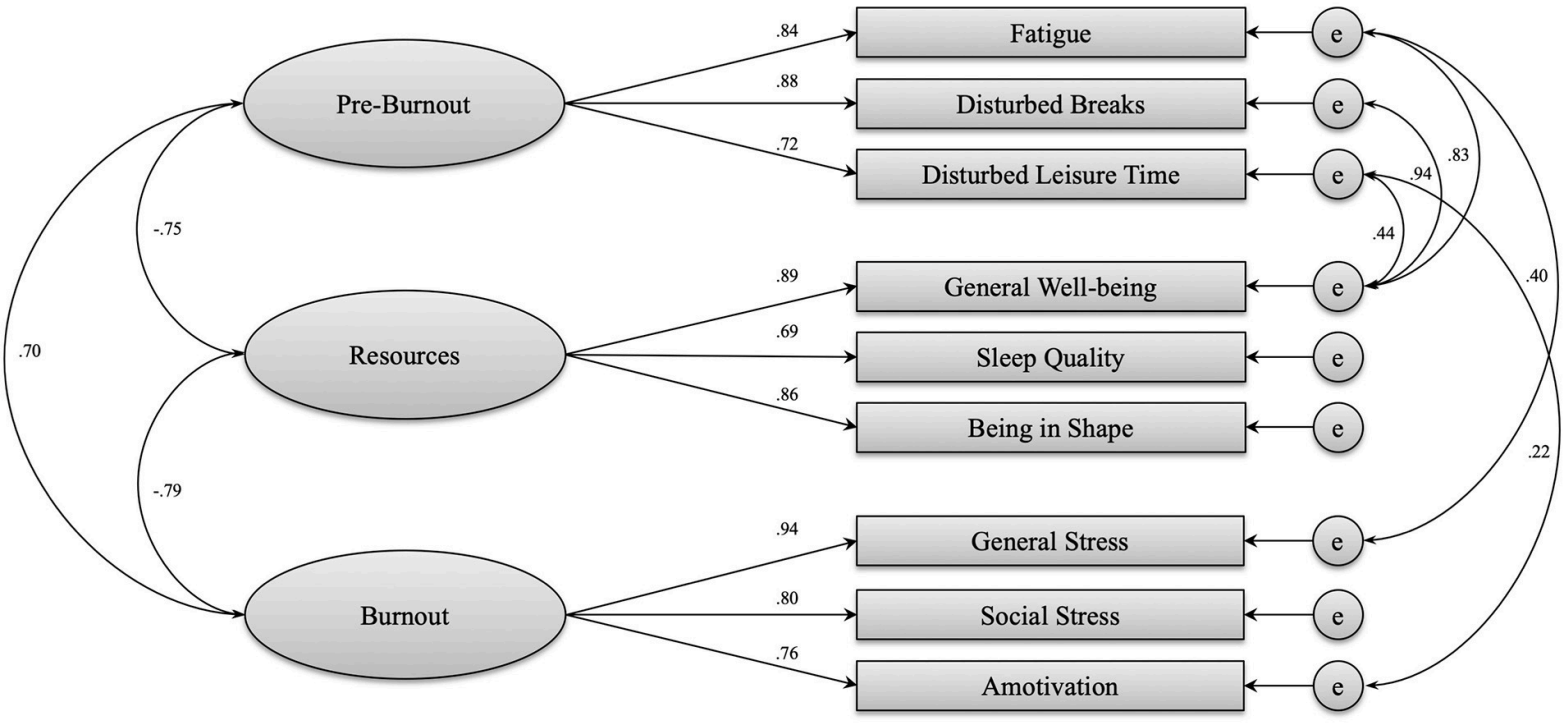
Structural model of the BPQ-C scales (χ^2^ = 96.898, *df* = 19, *p* < 0.001, CFI = 0.973, SRMR = 0.044, RMSEA = 0.093, LO90 = 0.075, HI90 = 0.112).

In a modified model, five specific correlations between the factors must be allowed to obtain a good structural model for the three factor model (Figure 1). This procedure was justified by assuming a high degree of similarity in the scale content. The modified model showed an improved fit (χ^2^ = 96.898, *df* = 19, *p* < 0.001, CFI = 0.973, SRMR = 0.044, RMSEA = 0.093, LO90 = 0.075, HI90 = 0.112) and all standardized factor loadings could be characterized as adequate and significant, ranging from 0.69 to 0.94. However, the RMSEA missed the threshold of 0.08. On the one hand, one reason for that could be the high proximity between scales of different factors, which already led to the assumption of a one-factorial structure in study 1. On the other hand, Kenny et al. (2014) confirm that the RMSEA should not be overestimated as a criterion in studies of low degrees of freedom. Moreover, Browne and Cudeck (1993) postulate a RSMEA of higher than 0.08 and smaller than 0.10 as a marginal fit.

The interscale correlations were examined as an indicator of construct validity. All scales within each factor correlated positively with the scales of the related factor (Table 8). Moreover, factor correlations were stable across the two samples. The results support the assumption that the strength and direction of all relationships were consistent with study 1. Furthermore, inter-factor correlations were examined within study 2. Pre-Burnout showed a moderate positive correlation with Burnout (*r* = 0.61) and a moderate negative correlation with Resources (*r* = −0.56), whereas Resources showed a moderate negative correlation with Burnout (*r* = −0.68).

**Table 8:** Intercorrelations for the samples of study 1 (*N* = 233) and study 2 (*N* = 473).

		**Scale**	**1**	**2**	**3**	**4**	**5**	**6**	**7**	**8**	**9**
**UPPER DATA MATRIX: STUDY 1**											
Pre-Burnout	1	Fatigue		0.64	0.59	−0.46	−0.66	−0.59	0.62	0.52	0.34
	2	Disturbed Breaks	**0.71**		0.67	−0.44	−0.58	−0.55	0.58	0.45	0.32
	3	Disturbed Leisure Time	0.59	0.67		−0.40	−0.50	−0.51	0.53	0.53	0.35
Resources	4	General Well–being	−0.42	−0.39	−0.27		0.65	**0.71**	−0.67	−0.49	−0.48
	5	Sleep Quality	−0.52	−0.43	−0.26	0.62		0.66	−0.63	−0.45	−0.34
	6	Being in Shape	−0.60	−0.56	−0.42	**0.77**	0.60		−0.60	−0.40	−0.45
Burnout	7	General Stress	0.64	0.58	0.38	−0.68	−0.62	−0.62		**0.71**	0.54
	8	Social Stress	0.50	0.53	0.33	−0.54	−0.49	−0.47	**0.75**		0.43
	9	Amotivation	0.43	0.49	0.42	−0.52	−0.41	−0.47	**0.70**	0.62	
**LOWER DATA MATRIX: STUDY 2**											

*r ≥ 0.70 are printed in bold type*.

Criterion validity of the BPQ-C was evaluated by examining the relationship between the BPQ-C and the MBI-C. As expected, the scales within the factors Burnout and Pre-Burnout correlated positively with *Emotional Exhaustion* and *Depersonalization* of the MBI-C (Table 9). Negative correlations occurred between these scales and *Personal Accomplishment*. For the scales within the factor Pre-Burnout, opposing relationships were shown. A positive relationship with *Personal Accomplishment* and a negative relationship with *Emotional Exhaustion* and *Depersonalization* manifested. Overall, the pattern of relationships between the scales underlines the validity of the BPQ-C. Internal consistency analyses revealed that all scales ranged above the critical value of α = 0.70 (α = 0.77–0.90). Identically, reliability for the three factors (range α = 0.82–0.94) can be assumed.

**Table 9:** Correlation between BPQ-C and MBI-C for the sample of study 2.

		**BPQ-C**	**Cronbach α**			**MBI-C**		
				**LO95**	**HI95**	**Emotional Exhaustion**	**Depersonalization**	**Personal Accomplishment**
Pre-Burnout	1	Fatigue	0.85	0.82	0.87	0.65	0.27	−0.19
	2	Disturbed Breaks	0.82	0.79	0.84	0.57	0.30	*n*.*s*.
	3	Disturbed Leisure Time	0.77	0.73	0.80	0.46	0.23	*n*.*s*.
Resources	4	General Well-being	0.90	0.88	0.91	−0.55	−0.34	0.43
	5	Sleep Quality	0.88	0.86	0.90	−0.53	−0.24	0.28
	6	Being in Shape	0.86	0.83	0.88	−0.60	−0.29	0.41
Burnout	7	General Stress	0.87	0.85	0.89	0.74	0.41	−0.30
	8	Social Stress	0.88	0.86	0.90	0.62	0.44	−0.26
	9	Amotivation	0.86	0.84	0.88	0.65	0.46	−0.26

*N = 473; LO95, lower limit of the 95%-confidence interval; HI95, upper limit of the 95%-confidence interval*.

## General Discussion

The aim of our two studies consisted in the development and validation of the BPQ-C as an instrument to assess risk factors of burnout in coaches. Considering internal consistency parameters, in both studies all BPQ-C scales and factors revealed good to excellent values of Cronbach's alpha. In study 1, the Exploratory Factor Analysis showed high loadings of each scale on the determined factor. However, several side loadings of more than |0.40| as well as the moderate intercorrelations of some scales between the BPQ-C factors (Table 8) indicated that the three factors of the BPQ-C are interrelated. These results were somehow expectable, as most of the scales originate from the RESTQ-Basic and RESTQ-Coach, therefore stemming from overlapping constructs. This assumption is supported by the CFA in study 2. Factor loadings between the latent variables as well as the specific correlations between the factors illustrated content-related proximity of the constructs. Therefore, results of the modified CFA model displayed a very good overall fit and supported the structure found in the Exploratory Factor Analysis of study 1. Taken together, these studies have provided substantial evidence for the reliability and validity of the BPQ-C as an innovative screening instrument. The BPQ-C may emerge as a valuable tool in understanding influencing factors for burnout in coaches. The BPQ-C should primarily be implemented to detect potential causes of burnout to derive individual preventive measures. It may serve as a validated and economic tool for the practical use in sports contexts.

The three factors of the BPQ-C cover a wide range of aspects that have been associated with burnout in recent research. In theory, the factor Pre-Burnout administers facets which may contribute to the development of burnout in the long-term. *Disturbed Breaks* and *Disturbed Leisure Time* deal with recovery deficits, interrupted recovery, and situational aspects that interrupt periods of rest (Kellmann et al., 2016b). Breaks not only serve as a compensatory purpose, but may also have preventive effects by means of recovery, nutrition, and coping with fatigue (Pelka et al., 2017). Timing, duration, and the context determine the effectiveness of each break. Kallus (2016b) postulates an increased susceptibility for disturbances, annoyances, and irritation during periods of recovery. The moderate to high correlations within the factor Pre-Burnout (Table 3) also indicate a direct relationship between disturbed periods of recovery (*Disturbed Breaks*/*Disturbed Leisure Time*) and the manifestation of physical symptoms (*Fatigue*). Therefore, Kallus (2016a) suggests that *Fatigue* may arise as a result of time pressure in job and training, being constantly disturbed during important work or breaks and a lack of sleep.

The factor Resources predominantly entails protective aspects with regard to the coaches' robustness toward burnout. In this context, *Being in Shape* determines the frequency of activities a coach approaches in order to maintain or increase his personal fitness. Mallett (2010) indicates that the majority of coaches had previously been engaged in sports as athletes but significantly reduced their physical activity after ending their career. Due to the high physical loads during their active time, coaches barely consider physical activity as a recovery strategy (Kellmann et al., 2016a). *Sleep Quality* may be considered as a protective but at the same time as a risk factor of burnout. On the one hand, sleep characterizes one of the most important and effective recovery strategies (Gerber et al., 2013). On the other hand, a lack of sleep together with a reduced sleep quality ranges among the main risk factors for burnout development (Söderström et al., 2012; Prather et al., 2013). As a summary of various recovery strategies *General Well-being* quantifies the general mood state and level of relaxation of a coach (Kallus, 2016a).

Unsurprisingly, the strongest associations appear between the burnout symptoms as proposed by Maslach et al. (1996) and the factor Burnout (Table 4). This is corroborated by several studies. For instance, *Social Stress* encompassed the frequency of conflicts in the social environment. Numerous authors have found that social stress is accompanied by increased values of burnout (Raedeke et al., 2000; Raedeke, 2004; Mazerolle et al., 2008; Altfeld and Kellmann, 2015). While the preceding scale represents specific stress reactions, *General Stress* measures unspecific stress reactions as a consequence of intense and discouraging episodes at work (Kallus, 2016a). In addition, the results of Bentzen et al. (2015) as well as McLean et al. (2012) indicate that *Amotivation* appears to be closely related to the development of burnout. The authors refer to the SDT (Ryan and Deci, 2002) to better understand the motivational process leading to burnout. With regard to the SDT, self-determined or autonomous motivation is substantiated by the satisfaction of three basic psychological needs (autonomy, competence, relatedness). *Amotivation* describes a lack of motivation in case these three psychological needs remain unsatisfied.

In summary, the BPQ-C covers a wide range of existing protective and risk factors for burnout within a single questionnaire. It can be used for the early detection of critical conditions and situations in order to initiate appropriate interventions at an early stage. While the factor Burnout administers those aspects that are directly linked to the development of burnout, the factor Resources encompasses predictive aspects for burnout, thereby exhibiting a health-oriented function. In contrast, Pre-Burnout describes situations and their consequences related to a disturbed recovery process. Pre-Burnout shows a close relationship to resources, which is highlighted by a number of specific correlations between these factors (Figure 1). Interventions for burnout prevention should therefore aim at the activation of individual resources and the avoidance of situations which might interfere with recovery. This is also supported by Awa et al. (2010) who concluded in their review that a combination of person-directed and organizational-directed interventions appears to be the most effective way to deal with burnout.

## Limitations and Future Research

Although we consider the results to be encouraging, further assessment of the validity of the BPQ-C is necessary. First, the cross-sectional design applied in both studies restricts the validity of the results. Therefore, longitudinal studies are essential to further evaluate the BPQ-C and establish the scales' test-retest reliability. Second, despite the BPQ-C's targeting of a representative sample of coaches, the demographic data insufficiently mirrors coaches in professional sports, especially in team sports. Future studies should scrutinize the psychometric properties of the BPQ-C with a larger unspecific as well as sport-specific sample. Third, the current studies were conducted with German coaches exclusively. To further explore the validity of the BPQ-C, cross-cultural coaches should participate in future studies.

## Conclusion

In summary, these two studies delineate the development and examination of a measure of risk factors for burnout in coaches for the first time. However, final decisions about the BPQ-C in accordance with theoretical underpinnings require extensive research and replication. The BPQ-C offers important information which is necessary to identify critical areas in the coaches' environment and the subsequent implementation of strategies for the prevention of burnout.

## Ethics Statement

The study was approved by the ethics committee of the psychological faculty of the Ruhr University Bochum (registration number: 284), and was completed according to the guidelines of the Declaration of Helsinki.

## Author Contributions

PS, JK, SA, CZ, and MK conceived and designed the studies. PS and MK recruited the participants. PS, JK, SA, CZ, KK, and MK analyzed the data. PS wrote the paper.

### Conflict of Interest Statement

The authors declare that the research was conducted in the absence of any commercial or financial relationships that could be construed as a potential conflict of interest.
